# Different Pattern in Circulating MicroRNA‐22‐3p Levels Between Patients With Primary Versus Secondary Sarcopenia

**DOI:** 10.1111/acel.70284

**Published:** 2025-11-14

**Authors:** Mirela Vatic, Anselm A. Derda, Tania Garfias‐Veitl, Ryosuke Sato, Goran Lončar, Guglielmo Fibbi, Wolfram Doehner, Christian Bär, Francesco Landi, Riccardo Calvani, Matteo Tosato, Roberto Bernabei, Emanuele Marzetti, Robert Kob, Cornel Sieber, Stefan D. Anker, Thomas Thum, Stephan von Haehling

**Affiliations:** ^1^ Department of Cardiology and Pneumology University Medical Center Göttingen Goettingen Germany; ^2^ German Center for Cardiovascular Research (DZHK) Partner Site Lower Saxony Germany; ^3^ Department of Cardiology and Angiology Hannover Medical School Hannover Germany; ^4^ Institute of Molecular and Translational Therapeutic Strategies (IMTTS) Hannover Medical School Hannover Germany; ^5^ Department of Medical Statistics University Medical Center Göttingen Göttingen Germany; ^6^ Dedinje Cardiovascular Institute Belgrade Serbia; ^7^ Faculty of Medicine University of Belgrade Belgrade Serbia; ^8^ Department of Geriatrics University Medical Center Göttingen Goettingen Germany; ^9^ Berlin Institute of Health Center for Regenerative Therapies Charité‐Universitätsmedizin Berlin Berlin Germany; ^10^ Deutsches Herzzentrum der Charité, Department of Cardiology‐Campus Virchow Charité Universitätsmedizin Berlin Berlin Germany; ^11^ German Center for Cardiovascular Research (DZHK), Partner Site Berlin Berlin Germany; ^12^ Center for Translational Regenerative Therapies Hannover Medical School Hannover Germany; ^13^ Department of Geriatrics, Orthopaedics and Rheumatology Università Cattolica del Sacro Cuore Rome Italy; ^14^ Fondazione Policlinico Universitario A. Gemelli IRCCS Rome Italy; ^15^ Institute for Biomedicine of Aging Friedrich‐Alexander‐Universität Erlangen‐Nürnberg Nuremberg Germany; ^16^ Division of Cardiology and Metabolism‐Heart Failure, Cachexia & Sarcopenia, Department of Cardiology (CVK) Charité University Medical Center Berlin Berlin Germany; ^17^ Institute of Heart Diseases Wroclaw Medical University Wroclaw Poland

**Keywords:** heart failure, sarcopenia, skeletal muscle

## Abstract

Sarcopenia, characterized by decreased skeletal muscle mass and strength, is classified as “primary” (due to aging) or “secondary” (due to diseases). MicroRNA‐22‐3p (miR‐22) regulates muscle differentiation and function. We assessed the diagnostic value of circulating miR‐22 levels in patients with primary and secondary sarcopenia. miR‐22 levels were evaluated in 61 older adults from the “Sarcopenia and Physical fRailty IN older people: multi‐componenT Treatment strategies” (SPRINTT) study and in 176 heart failure (HF) patients from the “Studies Investigating Co‐morbidities Aggravating HF” (SICA‐HF). miR‐22 expression profile was measured in serum by miR‐specific TaqMan quantitative real‐time PCR. In SPRINTT, 33 participants (54.1%) had primary sarcopenia. Subjects with primary sarcopenia had slower gait speed (0.7 [0.6–0.8] vs. 0.8 [0.7–1.0] m/s; *p* < 0.001) than those without sarcopenia. Multivariate analysis showed miR‐22 as an independent predictor of sarcopenia (adjusted OR 3.087, 95% CI 1.441–6.611, *p* = 0.004). In SICA‐HF, 28 patients (15.9%) had secondary sarcopenia. Sarcopenic HF patients were older (74.5 [68.7–80.2] vs. 68.4 [60.9–74.8] years; *p* = 0.001), had lower left ventricular ejection fraction (31.1 [26.2–47.5] vs. 40.0 [30.0–55.0] %; *p* = 0.025), lower handgrip strength (31.1 ± 6.0 vs. 37.0 ± 13.0 kg; *p* = 0.016) and lower absolute peak oxygen uptake (1181.3 ± 379.5 vs. 1593.0 ± 487.0 mL/min; *p* < 0.001) compared with those without sarcopenia. Multivariate logistic regression analysis showed miR‐22 as significantly associated with sarcopenia in HF patients (adjusted OR 0.409, 95% CI 0.193–0.867, *p* = 0.020). miR‐22 levels are significantly associated with both primary and secondary sarcopenia, suggesting its potential as a novel epigenetic biomarker of skeletal muscle dysfunction.

## Graphical abstract

Serum miR‐22 levels are significantly associated with both primary (age‐related) and secondary (heart failure‐related) sarcopenia and independently predict its presence. Circulating miR‐22 may be a novel, non‐invasive epigenetic biomarker for detecting sarcopenia in aging and heart failure.

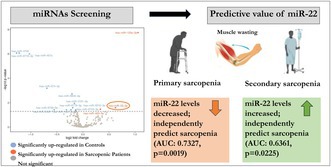

## 1 Introduction

Sarcopenia is a potentially treatable musculoskeletal disease that impacts activities of daily living and survival, and has attracted increasing clinical and research interest (von Haehling et al. 2020). Primary sarcopenia occurs when age‐related processes are the major drivers of muscle loss despite minor comorbidities, whereas secondary sarcopenia is predominantly driven by specific diseases, disuse, or malnutrition that amplify muscle decline beyond the usual effects of aging (Cruz‐Jentoft et al. 2019). This distinction, however, is more academic than clinical, as older adults with sarcopenia often exhibit overlapping causative factors, with age‐related processes still predominating in primary sarcopenia but frequently compounded by chronic illness, reduced physical activity, or nutritional deficits. With increasing life expectancy and a growing older population, the prevalence of sarcopenia is expected to rise significantly in the coming decades. This highlights the need for thorough research, analysis of the mechanisms underlying sarcopenia, and the search for screening biomarkers (von Haehling, Morley, and Anker 2010; Jensen 2008; Wiedmer et al. 2021; Conte et al. 2015).

Heart failure (HF) is a progressive clinical syndrome with an increasing number of affected patients, especially in Western countries. HF is associated with high morbidity, mortality, hospitalization rates, reduced quality of life, and economic burden, and its prognosis is highly dependent on comorbidities (Tomasoni et al. 2020). Studies have shown that 19.5%–47.3% of HF patients suffer from sarcopenia (Fülster et al. 2013; Canteri et al. 2019; Hajahmadi et al. 2017), and there is a growing understanding of the mechanisms mediating HF and sarcopenia (Sato et al. 2024).

MicroRNAs (miRNAs) are short non‐coding RNA molecules of approximately 22 nucleotides involved in the regulation of gene expression at the post‐transcriptional level (Thum et al. 2008; Kim et al. 2024). miRNAs have become one of the most promising targeting systems in the aetiopathogenesis of different diseases (Zhang et al. 2020; Vishnoi and Rani 2017; Wojciechowska et al. 2017; McGeary et al. 2019; Dowling et al. 2022). A recent phase 1b clinical study has investigated the safety and tolerability of a first‐in‐class miR‐132 inhibitor, an antisense oligonucleotide designed to reduce levels of miR‐132‐3p (miR‐132), in HF (Täubel et al. 2021). Indeed, miR‐132 is upregulated in HF and contributes to cardiomyocyte hypertrophy and is involved in pathological cardiac remodelling (Ucar et al. 2012). Interestingly, individuals with sarcopenia and chronic HF show notable alterations in plasma profiles of various miRNAs, including microRNA‐22‐3p (miR‐22) (Huang and Wang 2018) and microRNA‐423‐5p (miR‐423) (Tijsen et al. 2010). miR‐22 belongs to a broadly conserved miRNA family and is expressed and released by multiple tissues but predominantly by the myocardium, skeletal muscle, axons, and nerve terminals (Huang and Wang 2018). As an integral part of gene‐regulatory networks, miR‐22 targets several muscle function‐related genes, such as myotubularin‐related protein 2, myosin IXA, tropomyosin 3, or sarcalumenin (McGeary et al. 2019; Agarwal et al. 2015). Consistent with prior reports (Zhao et al. 2015), Yang et al. (2018) showed that miR‐22 plays an important role in skeletal muscle differentiation, vascular smooth muscle cells, and cardiac remodeling. These findings highlight miR‐22 as a promising candidate for potential diagnostic and therapeutic use. Previous studies have recognized miR‐22 as a key modulator of cardiac hypertrophy and a potent suppressor of cardiac autophagy in the context of aging (Gupta et al. 2016). Moreover, miR‐22 plays a role in the pathogenesis of HF by promoting hypertrophic growth and showing elevated expression levels in end‐stage disease. Growing evidence indicates that miR‐22 disrupts Ca^2+^ handling by impairing Ca^2+^ transients and sarcoplasmic reticulum Ca^2+^ uptake, ultimately contributing to contractile dysfunction and cardiac dilation (Gurha et al. 2013). MiR‐22 has also been identified as a modulator of skeletal muscle differentiation through its influence on the TGF‐β/SMAD signaling pathway (Singh et al. 2020). Wang and collaborators reported that elevated expression of miR‐22 suppresses the proliferation of skeletal muscle cells while enhancing their differentiation, whereas blocking miR‐22 produced reverse effects (Wang et al. 2022). Similarly, the downstream signaling pathway of miR‐22 has been found to be disrupted in the context of fibro‐adipogenic infiltration and muscle degeneration (Lin et al. 2020). Another preclinical study demonstrated that introducing a miR‐22 inhibitor into porcine satellite cells markedly reduced their differentiation, as evidenced by decreased levels of myogen and myosin heavy chain expression. Conversely, transfection with a miR‐22 mimic led to enhanced differentiation. Collectively, these results indicate that miR‐22 suppresses the proliferation of porcine satellite cells while promoting their differentiation (Dang et al. 2020). Using a preclinical model, Wen and colleagues confirmed the involvement of miR‐22 in regulating muscle fiber‐type transformation and investigated its mechanism in C2C12 myotubes. Their results suggest that miR‐22 influences fiber‐type switching by suppressing the AMPK/SIRT1/PGC‐1α signaling pathway (Wen et al. 2021). Additional studies in a mouse model have shown that exosomal miR‐22, which is elevated following circadian rhythm disturbances, contributes to the development of insulin resistance in skeletal muscle and may represent a promising biomarker and therapeutic target for metabolic muscle diseases (Zhang et al. 2023).

Therefore, we sought to evaluate the diagnostic potential of circulating miR‐22 for primary and secondary sarcopenia in a geriatric (older adults with physical frailty and sarcopenia from the SPRINTT study) and a HF population (from the SICA‐HF study).

## 2 Methods

### 2.1 Study Population: SPRINTT Study Subsample

To evaluate the diagnostic value of miR‐22 in primary sarcopenia, we conducted an analysis of 61 community‐dwelling individuals aged 70 years or older with physical frailty enrolled in the evaluator‐blinded, randomized controlled trial “Sarcopenia and Physical fRailty IN older people: multi‐componenT Treatment strategies” (SPRINTT) (Bernabei et al. 2022). The selection of participants was based on the presence of sarcopenia and physical frailty, irrespective of the presence of other chronic conditions (Landi et al. 2017). In addition, advanced cardiac disease states such as “Severe cardiovascular disease [NYHA class III or IV congestive HF, clinically significant valvular disease, history of cardiac arrest, presence of an implantable defibrillator, or uncontrolled angina]” have been explicitly excluded. This exclusion enhances the likelihood that the studied population reflects cases of primary sarcopenia more accurately. The objective of the SPRINTT study was to determine whether a multicomponent intervention comprising physical activity with technological support and nutritional counseling could prevent mobility disability in frail older adults with sarcopenia. The SPRINTT project was developed to establish a new, objective framework for defining physical frailty and sarcopenia, viewed as a pre‐disability state rooted in muscle failure. Its goals included identifying and characterizing individuals with physical frailty and sarcopenia who are at increased risk for negative health outcomes, and evaluating targeted interventions within this group (Bernabei et al. 2022). The trial was conducted in 16 clinical sites across 11 European countries between January 2016 and October 2019. In the SPRINTT project, physical frailty and sarcopenia were defined as the combination of reduced physical function and low appendicular lean mass (ALM) without mobility disability (Cesari et al. 2017). Physical function was evaluated using the Short Physical Performance Battery (SPPB), and participants with SPPB scores of 3–9 were considered eligible for the study (Cesari et al. 2017). Sarcopenia was defined as a low appendicular lean mass (ALM; < 19.75 kg in males and < 15.02 kg in females) or low ALM‐to‐body mass index (BMI) ratio (< 0.789 in males and < 0.512 in females) according to the criteria of the Foundation for the National Institutes of Health Sarcopenia Project (McLean et al. 2014). ALM was measured via whole‐body DEXA scan. The absence of mobility disability was operationalized as the ability to walk 400 m within 15 min without stopping for > 1 min, use of a walker, or help of another person. Participants were eligible for the SPRINTT study if they had a SPPB score between 3 and 9, were able to complete a 400‐m walk within 15 min without assistance or prolonged rest, and demonstrated low ALM, either using BMI‐adjusted ALM (< 0.789 in men and < 0.512 in women) or absolute ALM values (< 19.75 kg in men and < 15.02 kg in women). Additional inclusion criteria included the ability and willingness to provide informed consent, comply with study procedures, and accept randomization. Key exclusion criteria included severe cognitive impairment, current or recent cancer treatment, advanced cardiovascular or pulmonary disease (e.g., NYHA class III/IV HF, need for supplemental oxygen), severe osteoarthritis, progressive neurological disorders, end‐stage renal disease, recent major surgery or cardiovascular events, and ongoing participation in structured exercise or other interventional clinical trials. Individuals with medical or psychiatric conditions likely to interfere with participation, life expectancy under 12 months, or safety concerns during the baseline 400‐m test were also excluded (Bernabei et al. 2022). For the present study, we focused the analysis exclusively on a sample of participants with sarcopenia enrolled at the Institute for Biomedicine of Aging, Friedrich‐Alexander University of Erlangen‐Nuremberg.

Individuals who met all SPRINTT inclusion criteria but had an ALM either absolute or adjusted for BMI above the cut‐off were included as controls. Participants were classified as either cases (sarcopenic individuals) or controls (non‐sarcopenic but at risk), based on the variable “status.”

### 2.2 Study Population: SICA‐HF Cohort

A retrospective analysis of 176 ambulatory patients with HF from the Studies Investigating Co‐morbidities Aggravating HF (SICA‐HF), a prospective, multinational, observational study designed to assess the prevalence and impact of comorbidities in patients with chronic stable HF (von Haehling, Lainscak, et al. 2010), was conducted to assess the diagnostic potential of miR‐22 in secondary sarcopenia. Participants enrolled in the SICA‐HF study were required to have a clinical diagnosis of HF supported by objective evidence of cardiac dysfunction, such as a LVEF ≤ 40%, a left atrial dimension > 4.0 cm, or elevated natriuretic peptides (NT‐proBNP > 400 pg/mL or BNP > 150 pg/mL). Additional inclusion criteria included age over 18 years and the ability to provide informed consent. Key exclusion criteria were prior heart transplantation, recent major cardiovascular events or procedures (including myocardial infarction, unstable angina, stroke, revascularization, or abdominal surgery within the previous 6 weeks), ongoing dialysis, current pregnancy, or inability to understand the study procedures or provide informed consent (von Haehling, Lainscak, et al. 2010). Of the patients enrolled in SICA‐HF, we limited the present analysis to those enrolled at Charité Medical School Berlin, Germany, between January 2010 and March 2014. The diagnosis of sarcopenia was based on a reduction in appendicular skeletal muscle mass (ASM) index, that is, ASM adjusted for height squared, with the following revised consensus definition: ASM index < 7.26 kg/m^2^ in males and < 5.45 kg/m^2^ in females (Cruz‐Jentoft et al. 2019). ASM index is an anthropometric equation for predicting appendicular skeletal mass, calculated as ASM (kg) divided by height squared (m^2^), with sarcopenia defined as values below two standard deviations from young reference group mean. Body composition was assessed by DEXA. Exercise capacity was examined by a cardiopulmonary exercise test and a 6‐min walk test (6‐MWT).

### 2.3 Microarray Screening by Hummingbird Diagnostics

Initial screening of potential candidate miRNAs was performed by Hummingbird Diagnostics (Heidelberg, Germany) in serum samples of 32 participants with sarcopenia and 30 non‐sarcopenic controls. All incoming samples underwent a comprehensive quality check using the Agilent 2100 Bioanalyzer instrument (Santa Clara, United States of America) and the SmallRNA Kit according to HBDx SOP A‐0103. MiRNA samples that passed the RNA Quality Control were measured on an Agilent's Microarray Platform (Microarray Scanner SureScan Dx G5761A). MiRBase‐Chip (Human miRNA Microarray Release 21.0, 8 × 60K) was used to detect the miRNAs. A total of 2549 miRNAs were measurable, and we screened them for their potential as diagnostic markers for the diagnosis of sarcopenia. Upon further analysis, 275 miRNAs were detected in at least 75% of the samples of at least one group (sarcopenic, control). In the last step, we identified several miRNAs that surfaced as potential candidates. Some of the candidates were highly expressed in sarcopenic participants, in detail miR‐22‐3p, miR‐423‐5p, and miR‐125a‐3p. Biomarkers that were highly expressed in controls were: miR‐4730, miR‐6716‐3p, miR‐451, miR‐574‐3p, miR‐6834‐3p, miR‐3151‐3p, miR‐6779‐3p, miR‐5010‐3p, miR‐6739‐5p, miR‐6743‐3p, miR‐8485, miR‐483‐3p, among others. miR‐22 was selected for investigation based on prior reports demonstrating its involvement in skeletal muscle atrophy and wasting, making it a relevant target for our study (Huang and Wang 2018; Yang et al. 2018; Lin et al. 2020).

### 2.4 RNA Isolation and Heparinase Treatment

RNA was extracted from 100 μL serum using the miRNeasy Serum/Plasma Advanced Kit (Qiagen, Germantown, United States of America). All steps were performed according to the manufacturer's protocol. Synthetic cel‐miR‐39‐3p (1.6 × 108 copies/μL) was added for normalization and quality examination after quantitative real‐time polymerase chain reaction (qRT‐PCR) (Derda et al. 2015). In the final step of the isolation process, RNA was eluted with 30 μL of water. Treatment with heparinase was applied to avoid possible interactions of the RNA with traces of heparin (Garg et al. 2021).

### 2.5 Reverse Transcription and Real‐Time PCR

According to the manufacturer's instructions, the isolated RNA was transcribed into complementary DNA (cDNA) by reverse transcription using the TaqMan MicroRNA Reverse Transcription Kit (Applied Biosystems). Specific TaqMan miR assays (Applied Biosystems) for miR‐22‐3p, miR‐423‐5p, and miR‐6756‐3p were used to perform qRT‐PCR and measured using a Viia7 system. *Caenorhabditis elegans* cel‐miR‐39 was used for miR normalization. Cel‐miR‐39 was added to each sample to normalize the CT values and manage sample‐to‐sample variations (Madadi and Soleimani 2019). The 2‐ΔCq method (ΔCq = Cq[miR] − Cq[Cel‐miR‐39]) was used to calculate relative miR concentrations. Afterwards, data were scaled with log2 transformation (Derda et al. 2015; Garg et al. 2021).

### 2.6 Statistical Analysis

Continuous variables are presented as mean ± standard deviation (SD) for normally distributed data. For non‐normally distributed data, median with interquartile range (IQR) is reported. The normality of the distribution of continuous variables was tested by the Shapiro–Wilk test. As appropriate, differences between the sarcopenia and non‐sarcopenia groups were analyzed using the Student's unpaired *t*‐test or Mann–Whitney *U* test. Categorical data were analyzed using *χ* ^2^ tests. Univariate and multivariate logistic regression analyses were used to identify variables associated with sarcopenia, with the results expressed as odds ratio (OR) and 95% confidence interval (CI). The Youden index was used to assess the sensitivity and specificity of miR‐22 as a biomarker for sarcopenia. A two‐tailed *p*‐value of < 0.05 was considered statistically significant. All statistical analyses were performed using the Statistical Package for the Social Sciences (SPSS), version 29.0. Graphical illustration was performed using Graph Pad Prism Version 9.

### 2.7 MiR‐22 Target Analysis and Pathway Enrichment

To investigate the functional relevance of miR‐22 and mechanisms of miR‐22 changes in sarcopenia, we performed an in silico analysis of its experimentally validated mRNA targets and assessed their enrichment in biological pathways. Experimentally validated mRNA targets of miR‐22 were retrieved using the multiMiR R package (v1.30.0). The Kyoto Encyclopaedia of Gene and Genomes (KEGG) pathway enrichment analysis was performed with clusterProfiler (v4.16.0) and org.Hs.eg.db (v3.21.0), applying the Benjamini‐Hochberg method for multiple testing correction (*q* < 0.05 was considered statistically significant). All analyses were carried out in R (v4.5.0), and visualizations were generated with ggplot2 (v3.5.2).

## 3 Results

### 3.1 SPRINTT Substudy Cohort

#### 3.1.1 Baseline Clinical Characteristics

In the SPRINTT study, 54.1% (*n* = 33) participants had primary sarcopenia, with no difference in age, sex, BMI, or the prevalence of comorbidities other than history of cancer (27.3 vs. 7.1%, *p* = 0.042) between sarcopenic and non‐sarcopenic participants (Table 1). Those with sarcopenia had slower gait speed (0.7 [0.6–0.8] vs. 0.8 [0.7–1.0] m/s; *p* < 0.001) compared to participants with no sarcopenia.

**TABLE 1:** Baseline characteristics of participants with primary sarcopenia and non‐sarcopenic participants in the SPRINTT cohort.

	Non‐sarcopenic participants (*n* = 28)	Primary sarcopenia participants (*n* = 33)	*p*‐value
Age (years)	79.5 [77.2–82.0]	80.0 [76.0–84.0]	1.0
Women (%)	82.1	84.8	0.8
BMI (kg/m^2^)	29.7 ± 4.0	30.1 ± 6.6	0.8
Waist circumference (cm)	98.4 ± 10.7	100.3 ± 15.7	0.6
Hip circumference (cm)	105.6 [101.5–112.6]	105.5 [99.5–115.6]	0.9
Waist‐to‐hip ratio	0.9 [0.9–1.0]	0.9 [0.8–1.0]	0.4
HR (bpm)	70.4 ± 9.9	72.8 ± 9.2	0.3
Systolic BP (mmHg)	140.9 ± 20.7	145.8 ± 24.1	0.1
Diastolic BP (mmHg)	79.5 [72.2–84.7]	79.0 [75.0–90.0]	0.7
Number of falls in the last year	2 [1–2]	2 [1–2]	0.2
SARC‐F‐SCORE	4 [4–5]	4 [3–5]	**0.013**
SARC‐F‐cutoff ≥ 4, %	89.3	57.6	**0.006**
Gait speed (m/s)	0.8 [0.7–1.0]	0.7 [0.6–0.8]	**< 0.001**
Time to complete 5 stands (s)	17.2 [15.0–20.0]	16.4 [13.1–20.1]	0.3
Summary SPPB score (points)	8.0 [7.0–9.0]	7.0 [5.5–9.0]	0.2
ALM (kg)	20.1 [17.4–23.2]	14.9 [14.4–19.3]	**< 0.001**
ALMI (kg/m^2^)	7.4 [6.9–8.5]	6.3 [5.7–7.7]	**0.002**
ALM/BMI	0.6 [0.5–0.8]	0.5 [0.5–0.6]	**< 0.001**
ALM (kg), women, *n* = 51	18.5 [17.2–21.6]	14.8 [14.2–16.2]	**< 0.001**
ALMI (kg/m^2^), women, *n* = 51	7.2 [6.6–8.3]	6.2 [5.7–6.9]	**0.001**
ALM/BMI, women, *n* = 51	0.6 [0.5–0.7]	0.5 [0.4–0.6]	**< 0.001**
ALM (kg), men, *n* = 10	25.4 ± 3.3	22.5 ± 4.6	0.3
ALMI (kg/m^2^), men, *n* = 10	8.2 ± 0.8	7.9 ± 1.4	0.7
ALM/BMI, men, *n* = 10	0.9 ± 0.1	0.6 ± 0.1	**0.003**
Laboratory parameters			
Red blood cells (10^12^/L)	4.6 ± 0.4	4.6 ± 0.4	0.7
Hemoglobin (g/dL)	13.9 ± 1.2	13.7 ± 1.3	0.9
Hematocrit (%)	41.5 [40.0–43.0]	41.0 [38.0–44.0]	0.7
Platelet count (10^9^/L)	230.2 ± 59.5	232.8 ± 54.5	0.9
White blood cells (10^9^/L)	5.8 ± 1.5	6.7 ± 1.6	**0.020**
Neutrophils (10^9^/L)	3.5 ± 0.9	4.4 ± 1.3	**0.002**
Lymphocytes (10^9^/L)	1.3 [1.2–1.7]	1.4 [1.1–2.1]	0.9
Monocytes (10^9^/L)	0.5 ± 0.1	0.4 ± 0.1	0.2
Eosinophils (10^9^/L)	0.2 [0.1–0.3]	0.2 [0.1–0.2]	0.4
Basophils (10^9^/L)	0.04 [0.03–0.06]	0.03 [0.02–0.05]	0.3
Erythrocyte mean corpuscular volume (fl)	90.6 ± 3.9	90.1 ± 4.0	0.6
Erythrocyte mean corpuscular hemoglobin (pg)	30.3 ± 1.7	30.4 ± 1.7	0.8
Erythrocyte mean corpuscular hemoglobin concentration (g/dL)	33.5 [32.8–34.0]	33.8 [33.1–34.3]	0.1
Triglycerides (mg/dL)	111.0 [86.2–174.2]	123.0 [98.0–169.0]	0.4
Total cholesterol (mg/dL)	222.6 ± 48.5	251.4 ± 51.6	**0.030**
HDL cholesterol (mg/dL)	56.4 ± 12.5	66.1 ± 14.7	**0.008**
LDL cholesterol (mg/dL)	142.3 ± 37.8	161.6 ± 39.2	0.1
Albumin (g/dL)	4.0 ± 0.3	4.1 ± 0.3	0.1
Glucose (mg/dL)	96.0 [90.0–112.0]	98.0 [89.0–119.5]	0.6
Calcium (mg/dL)	9.4 [9.2–9.6]	9.5 [9.3–9.7]	0.3
Potassium (mmol/L)	4.3 [3.9–4.6]	4.2 [4.0–4.5]	0.9
Sodium (mmol/L)	142.5 [141.0–143.0]	139.0 [136.5–141.0]	**< 0.001**
Urea nitrogen (mg/dL)	15.5 [14.0–20.7]	17.0 [14.0–20.0]	0.8
Creatinine (mg/dL)	0.8 [0.7–0.8]	0.8 [0.6–1.0]	**0.022**
Aspartate aminotransferase (U/L)	25.5 [20.0–30.0]	25.0 [20.5–29.0]	0.8
Alanine aminotransferase (U/L)	19.5 [16.0–23.7]	18.0 [16.0–21.5]	0.4
Total protein (g/dL)	6.7 [6.5–7.0]	7.1 [6.7–7.4]	**0.001**
Vitamin D 25 (OH)D (ng/mL)	22.0 [16.0–31.5]	18.0 [12.0–21.5]	0.2
Comorbidities			
Hypertension (%)	75.0	72.7	0.8
Myocardial infarction (%)	0	9.1	0.1
HF (%)	10.7	21.2	0.3
Diabetes mellitus (%)	17.9	24.2	0.5
Transient ischemic attack or mini‐stroke (%)	14.3	12.1	0.9
Stroke (%)	3.6	12.1	0.4
History of cancer (%)	7.1	27.3	**0.042**
Previous hip fracture (%)	10.7	9.1	0.8
Weight loss > 3 kg during the last 6 months (%)	10.7	3.0	0.2

*Note:* The total gait speed was defined as the time for the faster of the two walks (if only 1 walk done, that time was recorded).

Abbreviations: ALM, appendicular lean mass; ALMI, ALM/height^2^ (kg/m^2^), appendicular lean mass index; BMI, Body Mass Index; HR, heart rate; SARC‐F, sarcopenia questionnaire (strength, assistance with walking, rise from a chair, climb stairs and falls); SPPB, short physical performance battery (scores range from 0 to 12, with lower scores indicating poorer physical function). Bold signifies = *p* < 0.05.

#### 3.1.2 miR‐22 Levels

**FIGURE 1:**
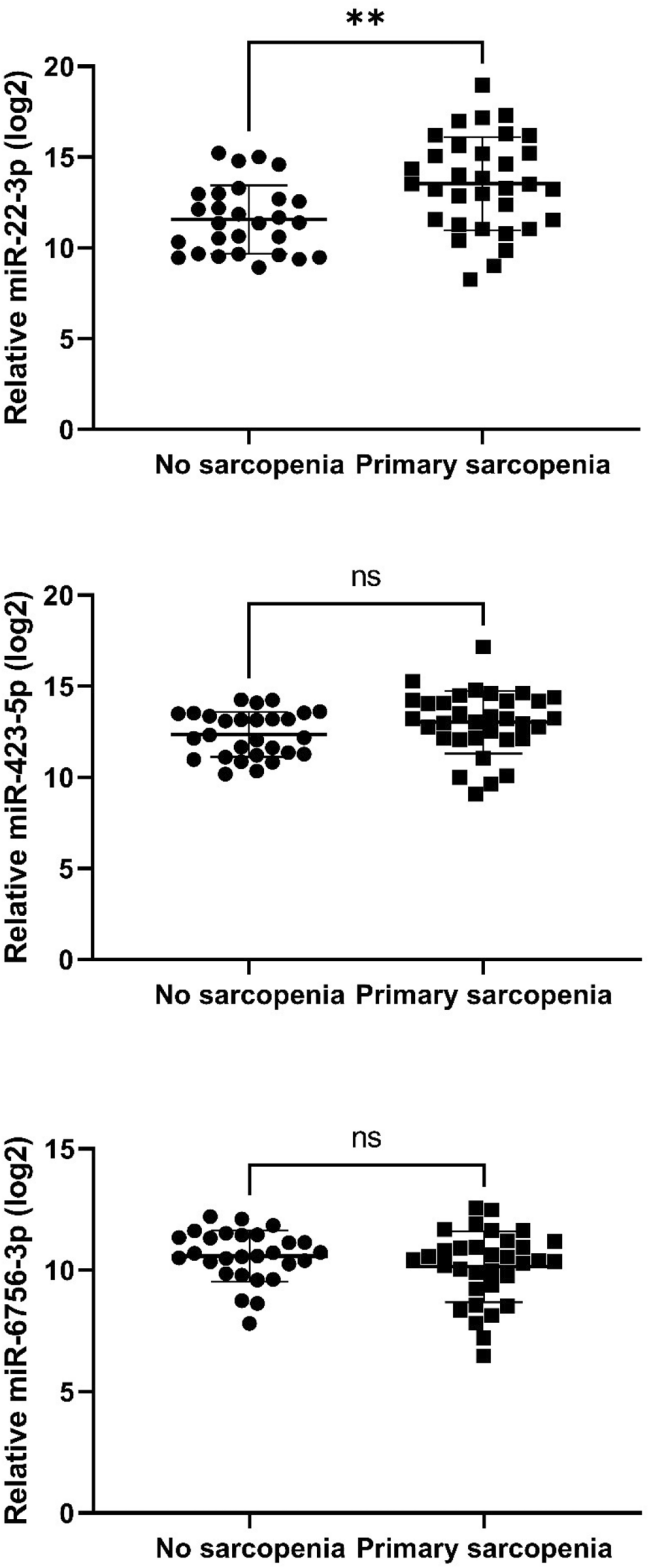
miRNAs (relative CT value)^#^ levels in non‐sarcopenic participants and those with primary sarcopenia. ^#^Relative CT‐value, log2 (2^ΔCT^ × 10000), where ΔCT = CT(miR‐22) − CT(cel‐39). ns, non‐significant. ** = *p* < 0.001.

**FIGURE 2:**
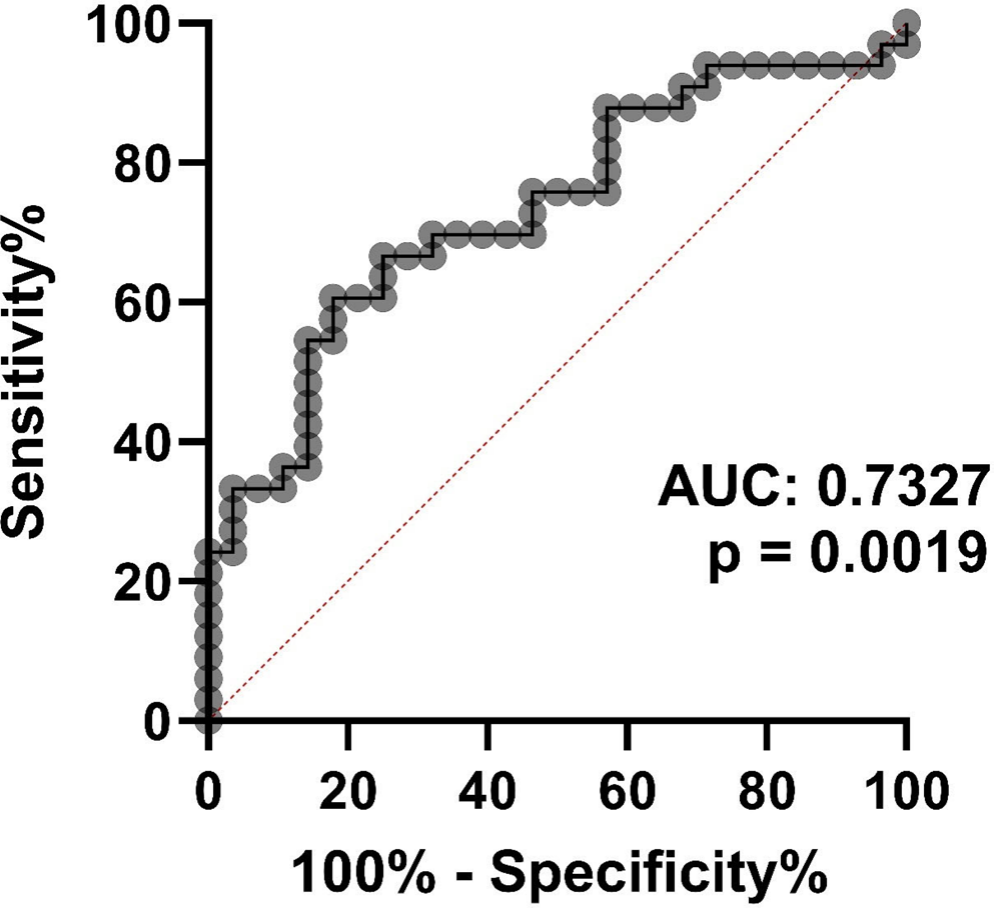
The discriminatory ability of miR‐22 in detecting sarcopenic participants in the SPRINTT cohort. A positive value is assigned to sarcopenic participants. AUC, area under the curve.

The qRT‐PCR analysis revealed that the serum levels of miR‐22 were significantly lower in sarcopenic participants than in those with no sarcopenia (13.5 ± 2.6 vs. 11.6 ± 1.9; *p* = 0.001) (Figure 1). No significant difference was found for miR‐423‐5p and miR‐6756‐3p (Figure 1). In a receiver operating characteristic (ROC) curve analysis, miR‐22 showed significant ability to discriminate between sarcopenic participants and those without sarcopenia (area under the curve 0.733, 95% CI [0.607–0.859], *p* = 0.002) (Figure 2). The discriminatory ability of miR‐22 in detecting sarcopenic individuals was high in the SPRINTT group, as a positive value was assigned to sarcopenic participants. The optimal cut‐off CT value of miR‐22 obtained from the Youden index predicting sarcopenia was 13.1, providing moderate sensitivity (60.6%) and high specificity (82.1%).

### 3.2 Associations Between miR‐22 Levels and Primary Sarcopenia

In univariate logistic regression analysis, miR‐22 levels were significantly associated with sarcopenia in the SPRINTT cohort (OR 1.471, 95% CI 1.136–1.903, *p* = 0.003) (Table 2). In the multivariate regression model adjusted for age, BMI, heart rate, and the presence of HF and previous cancer, miR‐22 levels (adjusted OR 3.087, 95% CI 1.441–6.611, *p* = 0.004) and miR‐423 levels (adjusted OR 2.268, 95% CI 0.084–0.850, *p* = 0.025) were significantly associated with primary sarcopenia.

**TABLE 2:** Univariate and multivariate logistic regression analysis predicting primary sarcopenia in the SPRINTT cohort.

SPRINTT cohort (*n* = 61)				
	Univariate		Multivariate	
	OR (95% CI)	*p*‐value	OR (95% CI)	*p*‐value
BMI (per 1 kg/m^2^ increase)	1.012 [0.923–1.109]	0.8	1.076 [0.949–1.220]	0.2
Heart failure	0.391 [0.096–1.584]	0.2	0.132 [0.017–1.001]	0.05
Heart rate (per 1 bpm increase)	1.028 [0.974–1.086]	0.3	1.014 [0.942–1.091]	0.7
Age (per 1 year increase)	1.006 [0.911–1.111]	0.9	1.005 [0.883–1.144]	0.9
miR‐22^a^	1.471 [1.136–1.903]	**0.003**	3.087 [1.441–6.611]	**0.004**
miR‐423^a^	1.351 [0.949–1.924]	0.1	0.268 [0.084–0.850]	**0.025**
History of cancer	2.667 [0.633–11.233]	0.2	6.877 [0.922–51.283]	0.1

Abbreviations: BMI, Body Mass Index; CI, confidence interval; OR, odds ratio.

^a^ Relative CT‐value, log2 (2^ΔCT^ × 10000), where ΔCT = CT(miR‐22) − CT(cel‐39). Bold signifies = *p* < 0.05.

#### 3.2.1 Correlations Between miR‐22 Levels and Selected Clinical Parameters

To investigate the potential involvement of miR‐22 in the clinical progression of the disease, we analyzed the relationship between its plasma levels and various clinical parameters. Our aim was to assess whether circulating miR‐22 might reflect underlying pathophysiological changes accompanying sarcopenia. We observed a weak to moderate positive correlation between circulating miR‐22 levels and heart rate (*R* = 0.252, *p* = 0.05). Likewise, miR‐22 levels correlated weakly with hemoglobin concentrations (*R* = 0.292, *p* = 0.02) and white blood cell count (*R* = 0.250, *p* = 0.05). Other clinical parameters analyzed did not show statistically significant correlations with circulating miR‐22.

### 3.3 SICA‐HF Cohort

#### 3.3.1 Baseline Clinical Characteristics

A total of 176 ambulatory stable patients with chronic HF (37 women) from the SICA‐HF were analyzed. Secondary sarcopenia was present in 28 (15.9%) patients. Baseline characteristics of sarcopenic and non‐sarcopenic HF patients are shown in Table 3. Sarcopenic HF patients were significantly older (74.5 [68.7–80.2] vs. 68.4 [60.9–74.8] years; *p* = 0.001), had a lower proportion of women (3.6 vs. 24.3%, *p* = 0.013), higher N‐terminal pro‐B‐type natriuretic peptide (NT‐proBNP) levels (3693.2 [1183.3–5000.0] vs. 1066.4 [521.3–2453.6] pg/mL; *p* < 0.001) and lower left ventricular ejection fraction (LVEF) (31.1 [26.2–47.5] vs. 40.0 [30.0–55.0] %; *p* = 0.025) compared to non‐sarcopenic HF patients. Diabetes mellitus (14.3 vs. 40.4%, *p* = 0.008) was less frequent in sarcopenic HF patients as compared to non‐sarcopenic HF patients. Instead, anemia was more frequent in patients with secondary sarcopenia compared to non‐sarcopenic counterparts (53.6 vs. 23.0%, *p* = 0.001).

**TABLE 3:** Baseline characteristics of patients with HF and with or without secondary sarcopenia in the SICA‐HF study.

	Non‐sarcopenic HF patients (*n* = 148)	Secondary sarcopenia in HF (*n* = 28)	*p*‐value
Age (years)	68.4 [60.9–74.8]	74.5 [68.7–80.2]	**0.001**
Women (%)	24.3	3.6	**0.013**
BMI (kg/m^2^)	29.2 [26.0–32.9]	25.1 [22.4–26.8]	**< 0.001**
Waist‐to‐hip ratio	0.98 [0.92–1.01]	0.96 [0.94–1.00]	0.9
NYHA class (1/2/3; %)	10.3/56.6/33.1	10.7/32.1/57.1	0.1
Peripheral oedema (%)	60.5	53.8	0.5
Coronary artery disease (%)	48.6	67.9	0.1
Atrial fibrillation (%)	35.8	46.4	0.3
Hypertension (%)	82.8	67.9	0.1
Diabetes mellitus (%)	40.4	14.3	**0.008**
Hyperlipidemia (%)	72.4	71.4	0.9
Anemia (%)	23.0	53.6	**0.001**
ACE‐I or ARB (%)	91.9	92.9	0.9
Beta blockers (%)	87.2	96.4	0.1
MRA (%)	40.5	32.1	0.4
Loop diuretics (%)	49.3	50.0	0.9
HR (bpm)	63.0 [58.0–72.0]	62.0 [58.0–69.0]	0.7
Systolic BP (mmHg)	125.0 [113.0–143.0]	134.0 [114.0–148.0]	0.3
Diastolic BP (mmHg)	75.0 [65.2–84.7]	71.0 [66.0–76.0]	0.2
Laboratory parameters			
Hemoglobin (g/dL)	13.0 ± 1.8	13.4 ± 1.0	**0.029**
Ferritin (μg/L)	152 ± 110.7	109 ± 49.5	0.1
Transferrin saturation (%)	16.4 ± 7.6	16.6 ± 4.4	0.4
Creatinine (mg/dL)	1.0 [0.9–1.2]	1.1 [1.1–1.2]	0.1
LDL cholesterol (mg/dL)	81.7 ± 25.1	73.0 ± 31.0	0.9
HDL cholesterol (mg/dL)	39.9 ± 9.2	39.3 ± 8.6	0.6
Triglycerides (mg/dL)	106.0 [78.0–144.0]	57.0 [48.0–100.0]	**0.031**
NT‐proBNP (pg/mL)	1066.4 [521.3–2453.6]	3693.2 [1183.3–5000.0]	**< 0.001**
CRP (mg/dL)	13.5 [10.4–25.0]	11.7 [8.4–25.0]	0.5
Echocardiographic parameters			
LVEF (%)	40.0.0 [30.9–55.0]	31.1 [26.2–47.5]	**0.025**
LA diameter (mm)	48.6 ± 4.2	48.3 ± 9.6	0.1
TAPSE (mm)	18.4 ± 4.7	14.0 ± 1.0	0.3
IVSd (mm)	12.0 [10.0–13.4]	12.0 [10.0–13.0]	0.8
LVPWd (mm)	11.0 [9.0–12.0]	9.1 [9.0–11.0]	**0.016**
LVIDd (mm)	55.6 ± 10.4	59.5 ± 8.6	**0.042**
Exercise haemodynamics			
Maximal systolic BP (mmHg)	155.0 [140.0–170.0]	147.0 [129.0–160.0]	0.1
Maximal diastolic BP (mmHg)	70.0 [60.0–80.0]	70.0 [60.0–80.0]	0.1
Maximal HR (bpm)	130.0 ± 22.0	118.0 ± 22.0	**0.007**
HR reserve (bpm)	67.0 ± 24.0	71.0 ± 15.0	**0.027**
Body composition			
Total fat mass (%)	34.5 ± 9.6	33.6 ± 6.6	0.2
Total fat mass (kg)	30.0 ± 11.0	25.6 ± 7.8	**0.007**
Lean mass (kg)	55.1 ± 11.6	49.7 ± 5.8	**0.006**
Lean arms (kg)	5.8 ± 1.5	5.4 ± 1.0	**0.037**
Lean legs (kg)	18.2 ± 3.9	15.3 ± 1.8	**< 0.001**
Lean trunk (kg)	27.3 ± 6.6	25.1 ± 3.1	0.1
Muscle strength			
Handgrip strength (kg)	37.0 ± 13.0	31.1 ± 6.0	**0.016**
Quadriceps strength (kg)	39.8 ± 13.6	33.0 ± 10.9	**0.014**
Exercise capacity			
Peak VO_2_ (mL/min/kg)	18.4 ± 4.6	14.9 ± 4.6	**0.001**
Absolute peak VO_2_ (mL/min)	1593.0 ± 487.0	1181.3 ± 379.5	**< 0.001**
6‐MWTD (m)	466.3 ± 120.0	389.0 ± 135.5	**0.009**
4‐m walk test (s)	3.8 [3.3–4.3]	4.1 [3.4–5.1]	0.1
SPPB total	12 [10–12]	11 [7.5–12]	**0.028**

Abbreviations: ACE‐I, angiotensin‐converting enzyme inhibitors; ARB, angiotensin receptor blockers; BMI, body mass index; BP, blood pressure; CRP, C‐reactive protein; HDL, high density lipoprotein; HDL, high‐density lipoprotein; IVSd (mm), interventricular septum thickness, measured during diastole; LA, left atrium; LDL, low density lipoprotein; LDL, low‐density lipoprotein; LVEF, left ventricular ejection fraction; LVIDd (mm), left ventricular internal dimension, diastole; LVPWd (mm), left ventricular posterior wall, diastole; MRA, mineralocorticoid receptor antagonist; NT‐proBNP, N‐terminal pro B‐type natriuretic peptide; NYHA, New York Heart Association; SPPB, short physical performance battery (scores range from 0 to 12, with lower scores indicating poorer physical function); TAPSE, tricuspid plane annular excursion; VO_2_, oxygen consumption; 6‐MWTD, 6‐min walking test distance. Bold signifies = *p* < 0.05.

Body composition analysis showed that the HF patients with predominantly secondary sarcopenia had significantly lower total lean mass (49.7 ± 5.8 vs. 55.1 ± 11.6 kg; *p* = 0.006), lower total fat mass (25.6 ± 7.8 vs. 30.0 ± 11.0 kg; *p* = 0.007), lower lean legs mass (15.3 ± 1.8 vs. 18.2 ± 3.9 kg; *p* < 0.001), and lower lean arms mass (5.4 ± 1.0 vs. 5.8 ± 1.5 kg; *p* = 0.037) than the non‐sarcopenic HF patients, as expected.

Regarding muscle strength and exercise capacity, sarcopenic HF patients had significantly lower handgrip strength (31.1 ± 6.0 vs. 37.0 ± 13.0 kg; *p* = 0.016), lower quadriceps strength (33.0 ± 10.9 vs. 39.8 ± 13.6 kg; *p* = 0.014), lower peak oxygen uptake (VO_2_) (14.9 ± 4.6 vs. 18.4 ± 4.6 mL/min/kg; *p* = 0.001), lower absolute peak VO_2_ (1181.3 ± 379.5 vs. 1593.0 ± 487.0 mL/min; *p* < 0.001), and shorter distances on the 6‐min walking test (389.0 ± 135.5 vs. 466.3 ± 120.0 m; *p* = 0.009) compared to non‐sarcopenic HF patients. No significant differences were observed in the 4‐m walk test between the two groups (4.1 [3.4–5.1] vs. 3.8 [3.3–4.3] sec; *p* = 0.090). The corresponding median gate speeds were 1.05 m/s for the non‐sarcopenic group vs. 0.98 m/s for HF patients with secondary sarcopenia. Sarcopenic HF patients also had significantly lower SPPB scores and more restricted mobility than those with non‐sarcopenia (11 [7.5–12] vs. 12 [10–12]; *p* = 0.028).

#### 3.3.2 miR‐22 Levels

**FIGURE 3:**
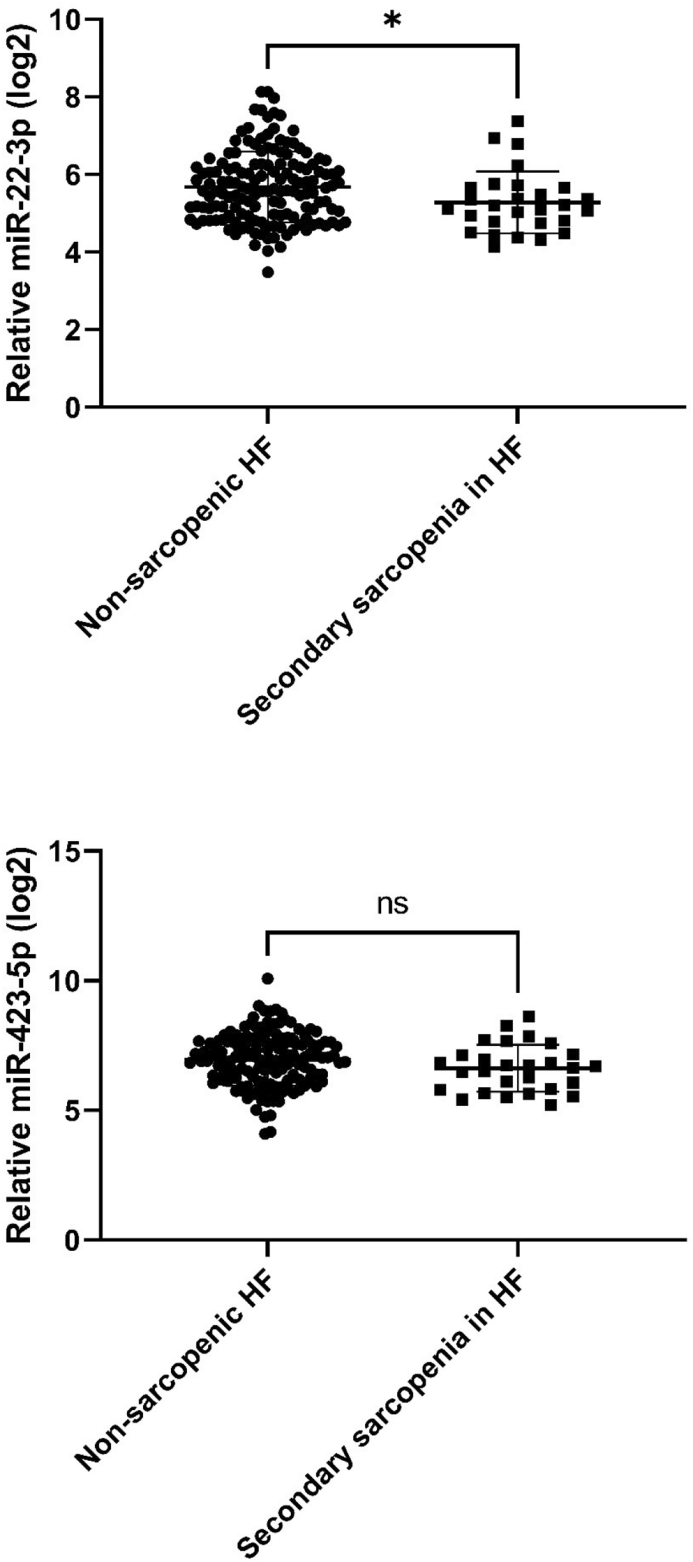
miRNAs (relative CT value)^#^ levels in the non‐sarcopenic HF patients and HF patients with secondary sarcopenia. ^#^Relative CT‐value, log2 (2^ΔCT^ × 10000), where ΔCT = CT(miR‐22) − CT(cel‐39). ns, non‐significant. * = *p* < 0.05.

**FIGURE 4:**
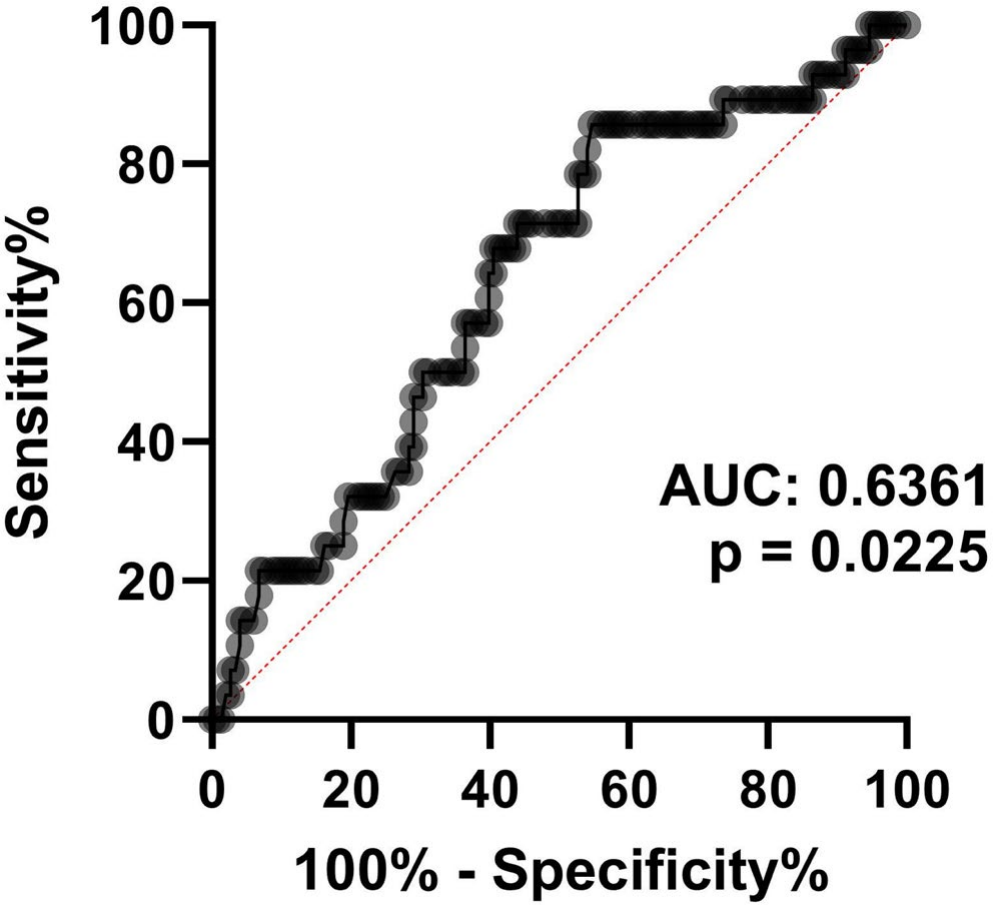
The discriminatory ability of miR‐22 in ruling out sarcopenic HF patients in the SICA‐HF study. A positive value is assigned to non‐sarcopenic patients. AUC, Area Under the Curve.

The qRT‐PCR analysis showed higher levels of miR‐22 in sarcopenic HF patients compared to those with no sarcopenia (5.2 ± 0.8 vs. 5.7 ± 0.9; *p* = 0.032), as displayed in Figure 3. No significant difference was detected for miR‐423‐5p (Figure 3). ROC curve analysis was performed to appraise the potential value of miR‐22 as a diagnostic marker of sarcopenia. Here, miR‐22 showed a significant ability to discriminate between sarcopenic HF patients and those without sarcopenia (area under the curve 0.636, 95% CI [0.522–0.746], *p* = 0.031), as shown in Figure 4. In the cohort of SICA‐HF, miR‐22 was able to rule out sarcopenic HF patients, as a positive value was assigned to non‐sarcopenic patients.

The optimal cut‐off CT value of miR‐22 obtained from the Youden index predicting sarcopenia was 5.39, providing high specificity (85.7%) and relatively low sensitivity (45.3%).

### 3.4 Associations Between miR‐22 Levels and Sarcopenia

In univariate logistic regression analysis, CT values of miR‐22 were significantly associated with sarcopenia in HF patients (OR 0.578, 95% CI 0.347–0.963, *p* = 0.035) (Table 4). In a multivariate regression model adjusted for age, diabetes mellitus, LVEF, NT‐proBNP levels, and absolute peak VO_2_ values, CT values of miR‐22 (adjusted OR 0.409, 95% CI 0.193–0.867, *p* = 0.020) and BMI (adjusted OR 0.784, 95% CI 0.642–0.958, *p* = 0.018) were still significantly associated with secondary sarcopenia in HF patients.

**TABLE 4:** Univariate and multivariate logistic regression analysis predicting secondary sarcopenia in the SICA‐HF cohort.

SICA‐HF cohort (*n* = 176)				
	Univariate		Multivariate	
	OR (95% CI)	*p*‐value	OR (95% CI)	*p*‐value
BMI (per 1 kg/m^2^ increase)	0.765 [0.673–0.869]	**< 0.001**	0.784 [0.642–0.958]	**0.018**
Diabetes mellitus	0.246 [0.081–0.745]	**0.013**	0.436 [0.110–1.720]	0.2
LVEF (per 10% increase)	0.695 [0.501–0.965]	**0.030**	0.981 [0.567–1.698]	0.9
Age (per 1 year increase)	1.079 [1.027–1.133]	**0.003**	1.067 [0.991–1.148]	0.1
miR‐22^a^	0.578 [0.347–0.963]	**0.035**	0.409 [0.193–0.867]	**0.020**
Log NT‐proBNP (per log pg/mL increase)	4.381 [1.938–9.905]	**< 0.001**	1.930 [0.444–8.388]	0.4
Absolute peakVO_2_ (per 1 SD increase)	0.998 [0.996–0.999]	**< 0.001**	0.570 [0.256–1.268]	0.2

Abbreviations: BMI, Body Mass Index; CI, confidence interval; LVEF, left ventricular ejection fraction; NT‐proBNP, N‐terminal pro B‐type natriuretic peptide; OR, odds ratio; VO_2_, oxygen consumption.

^a^ Relative CT‐value, log2 (2^ΔCT^ × 10000), where ΔCT = CT(miR‐22) − CT(cel‐39). Bold signifies = *p* < 0.05.

### 3.5 Correlations Between miR‐22 Levels and Left Ventricular Cardiac Hypertrophy

In the SICA‐HF sample, miR‐22 levels were moderately and negatively correlated with the interventricular septum diameter ([IVSd], [*R* = −0.554, *p* = 0.002]). In subgroup analyses of the HF patients with no or mild cardiac hypertrophy, miR‐22 levels showed an even stronger significant correlation with IVSd (*R* = −0.613, *p* = 0.002).

#### 3.5.1 Correlations Between miR‐22 Levels and Selected Clinical Parameters

To explore the potential role of miR‐22 in the clinical course of the disease, we examined correlations between its plasma levels and selected clinical parameters, aiming to assess whether circulating miR‐22 reflects relevant pathophysiological changes and could serve as a marker of disease severity or progression. Circulating levels of miR‐22 and miR‐423 correlated strongly (*R* = 0.858, *p* < 0.01), indicating that the expression patterns of these two microRNAs are closely aligned. We observed weak but statistically significant correlations between circulating miR‐22 levels and hemoglobin (*R* = 0.187, *p* = 0.01) and osteocalcin concentrations (*R* = −0.165, *p* = 0.03). Other clinical parameters analyzed did not show statistically significant correlations with circulating miR‐22.

### 3.6 Functional Annotation and Enrichment of miR‐22 Targets

**FIGURE 5:**
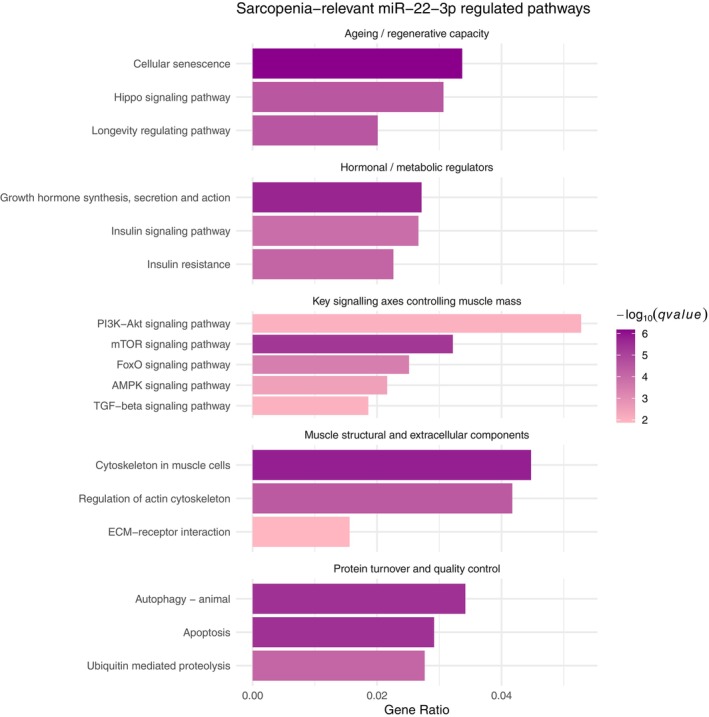
KEGG pathway enrichment of experimentally validated miR‐22 targets. Sarcopenia‐related pathways are ranked by gene ratio (*x*‐axis), defined as the proportion of significant genes relative to the total number of genes in each pathway. Pathways are colored by adjusted *p*‐value, with significance determined at *q* < 0.05 after Benjamini‐Hochberg correction.

Using in silico analysis, we found experimentally validated targets of miR‐22 grouped into five key functional categories including protein turnover and quality control, structural and extracellular matrix components, major signaling pathways regulating muscle mass, hormonal and metabolic regulation, and aging‐related regenerative capacity. Notably, the mechanistic target of rapamycin (mTOR), phosphoinositide 3‐kinase‐protein kinase B (PI3K‐Akt), AMP‐activated protein kinase (AMPK), forkhead box O (FoxO), and transforming growth factor beta (TGF‐β) pathways, central to muscle mass and protein metabolism, were enriched. Additional pathways involving ubiquitin‐mediated proteolysis, autophagy, and apoptosis suggest a role for miR‐22 in muscle protein degradation and cellular homeostasis. Enrichment in insulin signaling, growth hormone pathways, and longevity regulation further indicates miR‐22's involvement in systemic hormonal control and age‐related muscle decline (Figure 5).

## 4 Discussion

There are two key findings from this study. First, the prevalence of sarcopenia was high in the chronic stable HF cohort. Second, miR‐22 was significantly and inversely associated with both primary and secondary sarcopenia. The study is based on the existence of two different concepts of sarcopenia: primary, predominantly age‐dependent and secondary, predominantly associated with a specific disease (i.e., HF). To the best of our knowledge, this is the first study that evaluated the diagnostic value of miR‐22 for primary and secondary sarcopenia in two independent study populations.

The prevalence of secondary sarcopenia in SICA‐HF was high (15.9%), even though somewhat lower than that observed in chronic kidney disease (40%), diabetes mellitus (30%–40%), anemia (22%–37%) and chronic obstructive pulmonary disease (20.5%) (Paolillo et al. 2020; Manceau et al. 2022) confirming that sarcopenia is a frequent comorbidity in HF patients. Recent findings provide an attractive target, miR‐22, for diagnostic and possibly therapeutic applications in muscle and cardiac disease. miR‐22 is a conserved miRNA, which is highly expressed in cardiac and skeletal muscle (Zhao et al. 2015; Wang et al. 2022; Huang and Wang 2014). miR‐22 has been reported as a regulator of skeletal muscle differentiation via the transforming growth factor‐β (TGF‐β)/SMAD pathway (Singh et al. 2020). Wang et al. (2022). found that overexpression of miR‐22 inhibited proliferation of skeletal muscle cells and promoted its differentiation, whereas inhibition of miR‐22 resulted in the opposite. Similarly, the miR‐22 downstream pathway was shown to be dysregulated during fibro‐adipogenic fatty infiltration and muscle degeneration (Lin et al. 2020). On the other note, Gupta et al. (2016). identified miR‐22 as an important regulator of cardiac hypertrophy and a strong inhibitor of age‐related cardiac autophagy. Furthermore, miR‐22 is involved in the pathophysiology of HF, promotes cardiac dilation and is upregulated in end‐stage HF (Gurha et al. 2013). Increasing evidence suggests that miR‐22 impairs Ca^2+^ transient, Ca^2+^ loading into the sarcoplasmic reticulum, leading to contractile dysfunction and cardiac dilation (Gurha et al. 2013).

In the SPRINTT study of older adults with a low prevalence of at most mildly symptomatic HF, those with sarcopenia had a slower gait speed on the 4‐m walk test than those with no sarcopenia. On the other hand, the total SPPB score did not differ between the two groups, likely because a SPPB score of 3–9 was an eligibility criterion for the SPRINTT trial. Serum miR‐22 levels were significantly lower in participants with primary sarcopenia compared to those without sarcopenia. Figure 1 displays miRNA levels as relative cycle threshold (CT) values obtained from RT‐qPCR analysis, where a higher CT value indicates a lower absolute concentration of the biomarker miR‐22 in the blood. This finding implies that sarcopenic participants exhibited higher CT values, reflecting significantly lower serum miR‐22 levels compared to participants without primary sarcopenia. Moreover, miR‐22 was an independent predictor of sarcopenia, suggesting its potential as a biomarker of primary sarcopenia in the geriatric cohort. In the context of skeletal muscle, miR‐22 has been found to be involved in various biological processes, including muscle development, proliferation, and differentiation (Wang et al. 2022).

In contrast, HF patients with predominantly secondary sarcopenia due to HF had significantly higher serum levels of miR‐22 than non‐sarcopenic HF patients in the SICA‐HF cohort. As shown in Figure 3, a lower CT value in this context indicates that the target molecule—miR‐22—is present in higher concentrations, allowing it to be amplified earlier during the RT‐qPCR analysis. This highlights an inverse relationship between CT values and biomarker abundance, further supporting the differential expression patterns of miR‐22 in primary and secondary sarcopenia.

However, in a multivariate regression analysis, miR‐22 was again an independent and significant predictor of secondary sarcopenia. Circulating miR‐22 levels showed great potential as a biomarker for discriminating secondary sarcopenia in patients with HF on the ROC curve. In addition, patients with sarcopenic HF not only had reduced exercise capacity but also more severe HF, as reflected by lower LVEF and higher NT‐proBNP levels, compared to those without sarcopenia. These findings are consistent with previous reports showing that increased expression of miR‐22 exacerbates HF (Gurha et al. 2013). Similarly, we showed that miR‐22 levels correlated moderately with septal cardiac hypertrophy, again supporting previous findings (Tu et al. 2013).

In the SPRINTT study, circulating miR‐22 levels showed modest but noteworthy correlations with several clinical parameters. Specifically, miR‐22 levels were weakly to moderately associated with increased heart rate, hemoglobin concentration, and white blood cell count. These associations may reflect potential links between miR‐22 expression and physiological processes related to cardiovascular function, oxygen transport, and immune activity in an older population. Data from the SICA‐HF cohort revealed stronger correlations between miR‐22 levels and major clinical conditions. Taken together, these findings across both cohorts suggest that circulating miR‐22 may reflect a range of systemic processes, from subtle physiological shifts in older adults to more pronounced pathological conditions such as HF and cancer. This highlights its potential utility as a multifaceted biomarker in aging populations with comorbidities.

Our in silico pathway enrichment analysis based on experimentally validated miR‐22 targets indicates a broad involvement of miR‐22 in biological processes critical to muscle maintenance and degeneration. The enriched pathways clustered into five key domains: protein turnover and quality control, structural and extracellular matrix components, major signaling pathways regulating muscle homeostasis, hormonal and metabolic regulation, and cellular aging and regeneration (Figure 5). These categories mirror the pathophysiological hallmarks of sarcopenia and align with previous experimental evidence supporting miR‐22's role in muscle biology (Wang et al. 2022; Wen et al. 2021). In neonatal lambs, systemic inhibition of miR‐22 led to reduced expression in both circulation and skeletal muscles, accompanied by a shift toward oxidative fiber types and altered expression of histone deacetylase (HDAC) and sirtuin (SIRT) genes, key regulators of muscle metabolism and hypertrophy (Duckett et al. 2025). Notably, our analysis also identified SIRT1 within networks linked to senescence, longevity, FoxO, and AMPK signaling, reinforcing the relevance of these pathways. Key regulatory pathways enriched for miR‐22 targets include mTOR, PI3K‐Akt, AMPK, FoxO, and TGF‐β, which together govern muscle protein synthesis, degradation, and energy balance (Vainshtein and Sandri 2020; Sandri et al. 2004; Kjøbsted et al. 2018; Lan et al. 2024). This suggests that miR‐22 may modulate the balance between anabolic and catabolic states in muscle. Additional enrichment in pathways related to proteolysis, autophagy, and apoptosis further supports its role in muscle protein degradation and cellular quality control (Wang et al. 2022; Paez et al. 2023). The identification of insulin signaling, growth hormone pathways, and longevity regulation links miR‐22 to systemic endocrine and metabolic control relevant to both primary and secondary sarcopenia (Fujita et al. 2006; Bian et al. 2020). Moreover, its association with genes involved in extracellular matrix remodeling suggests a role in structural integrity and repair processes of aging muscle (Cai et al. 2023). This in silico analysis was designed to explore the mechanistic aspects of miR‐22 in the context of sarcopenia. It revealed a complex regulatory network through which miR‐22 may influence both muscle‐intrinsic and systemic processes contributing to sarcopenia pathophysiology.

Our study demonstrates the potential diagnostic value of miR‐22 for detecting primary sarcopenia in older adults with physical frailty and sarcopenia as well as secondary sarcopenia in HF patients. However, we found an inverse relationship between miR‐22 levels and primary versus secondary sarcopenia. This divergence likely reflects underlying biological and methodological factors. A plausible explanation for this discrepancy is the tissue‐specific origin of circulating miR‐22 (Sato and von Haehling 2023). Since the cases of primary sarcopenia in the SPRINTT study had a low prevalence of cardiac diseases, the changes in serum miR‐22 levels might be primarily derived from skeletal muscle, and therefore, the reduced expression of miR‐22 mainly could reflect a low skeletal muscle mass. Another possible explanation would be that miR‐22 is suppressed as a compensatory mechanism to maintain muscle mass, given that miR‐22 overexpression inhibits muscle proliferation (Wang et al. 2022). Its downregulation in sarcopenic patients could represent a compensatory mechanism to preserve muscle regeneration and counteract muscle atrophy. Therefore, reduced miR‐22 expression in individuals with primary sarcopenia may reflect an adaptive response aimed at maintaining muscle progenitor cell populations or enhancing anabolic signaling pathways that mitigate muscle wasting (Wang et al. 2022). In contrast, miR‐22 levels were elevated in cases of secondary sarcopenia due to HF in the SICA‐HF cohort, with these results being consistent with previous studies indicating that miR‐22 is upregulated in cardiac hypertrophy, pathological cardiac remodeling, and HF (Huang and Wang 2014). In this context, miR‐22 may be released predominantly from myocardial tissue, reflecting the ongoing cardiac disease process. Given that cardiomyocytes are known to actively secrete miRNAs into the circulation, the elevated miR‐22 levels observed in HF patients may, at least in part, reflect cardiac tissue damage or remodelling (Diez‐Cuñado et al. 2018). The upregulation of miR‐22 in these patients might therefore not primarily reflect skeletal muscle dysfunction, but rather represent a secondary systemic effect resulting from both sarcopenia and HF. The dual origin of miR‐22, cardiac versus skeletal muscle, may explain the inverse circulating miR‐22 levels observed in primary and secondary sarcopenia. However, given the fundamental physiological and morphological differences between cardiac and skeletal muscle, it remains unclear to what extent myocardial‐derived miR‐22 can influence overall serum miR‐22 levels and even outweigh skeletal muscle‐derived miR‐22 in all individuals. It is likely that the regulatory networks governing miR‐22 expression differ substantially between cardiac and skeletal muscle. Finally, we propose that the significant disparity in CT values for miR‐22, along with the observed inverse association between miR‐22 levels and primary and secondary sarcopenia in the SPRINTT and SICA‐HF cohorts, may be attributable to the well‐documented and pervasive influence of batch effects in high‐throughput data, as the samples from these cohorts were measured and sequenced at different time points. One of the key questions concerns whether elevated circulating levels of miR‐22 in sarcopenic patients reflect increased expression in skeletal muscle tissue. While miR‐22 is known to be abundantly expressed in muscle and involved in myogenesis and muscle homeostasis, direct evidence linking serum levels with skeletal muscle‐specific expression in sarcopenia remains limited. A few studies have demonstrated concordance between muscle‐derived and circulating miRNA profiles in other muscle‐related conditions, suggesting that muscle tissue may indeed contribute to serum miR‐22 levels (Coenen‐Stass et al. 2018). However, further research using paired muscle biopsy and serum samples in sarcopenic cohorts is needed to confirm this relationship.

Additionally, we note that the diagnostic criteria for sarcopenia differed between the two cohorts, as per the EWGSOP2 guidance that “tool selection may depend upon the patient.” This heterogeneity, combined with relatively small sample sizes and overlapping clinical features further complicates direct comparisons. Overall, our findings highlight the potential of miR‐22 as a biomarker for skeletal muscle dysfunction, while also underscoring the need for further large‐scale studies to dissect the source‐specific contributions of miR‐22 and to validate its role in both primary and secondary sarcopenia.

## 5 Study Limitations

Firstly, the diagnostic criteria for sarcopenia differ between the two cohorts, which aligns with the most recent version of the sarcopenia definition by the European Working Group on Sarcopenia in Older People (EWGSOP2) that suggests that “tool selection (for the diagnosis of sarcopenia) may depend upon the patient” (Cruz‐Jentoft et al. 2019). Secondly, the cohort sizes in both cohorts were relatively small. Additionally, the study examines two distinct concepts of sarcopenia: primary or age‐dependent sarcopenia, and secondary sarcopenia associated with HF. While we observed patterns predominantly related to age in the SPRINTT sub‐study (where 15% of participants had HF, including 21% of those with sarcopenia) and predominantly HF‐related characteristics in the SICA‐HF cohort, the overlap in demographic characteristics, including the presence of older individuals in the SICA‐HF cohort, suggests some degree of conceptual overlap. Finally, there might be a batch effect that explains the large difference in CT values and the relative amount of miR‐22 between SPRINTT and SICA‐HF, as these two cohorts were sequenced at different time points. The conditions and measuring protocols were not completely comparable, or another factor like HF pathology could be used to explain these differences. Further large‐scale studies are warranted to clarify the role of miR‐22 as a biomarker for the diagnosis of primary and secondary sarcopenia.

## 6 Conclusion

miR‐22 levels are significantly and inversely correlated with both primary sarcopenia in the geriatric cohort and secondary sarcopenia in the HF cohort. These results could be relevant in terms of new understanding of the disease and developing new diagnostic approaches and standards. Furthermore, miR‐22 may also represent a promising therapeutic target for future interventions.

## Author Contributions

**Stephan von Haehling** and **Thomas Thum:** conceptualization. **Mirela Vatic**, **Anselm A. Derda**, **Tania Garfias‐Veitl**, **Ryosuke Sato**, **Emanuele Marzetti**, **Thomas Thum**, and **Stephan von Haehling:** methodology. **Mirela Vatic**, **Anselm A. Derda**, **Tania Garfias‐Veitl**, and **Ryosuke Sato:** investigation. **Mirela Vatic**, **Anselm A. Derda**, and **Tania Garfias‐Veitl:** data analysis. **Mirela Vatic**, **Anselm A. Derda**, and **Ryosuke Sato:** visualization. **Stephan von Haehling:** funding acquisition. **Stephan von Haehling**, **Tania Garfias‐Veitl**, and **Thomas Thum:** supervision. **Mirela Vatic** and **Anselm A. Derda:** writing – original draft. **Mirela Vatic**, **Anselm A. Derda**, **Tania Garfias‐Veitl**, **Ryosuke Sato**, **Goran Lončar**, **Guglielmo Fibbi**, **Wolfram Doehner**, **Christian Bär**, **Francesco Landi**, **Riccardo Calvani**, **Matteo Tosato**, **Roberto Bernabei**, **Emanuele Marzetti**, **Robert Kob**, **Cornel Sieber**, **Stefan D. Anker**, **Thomas Thum**, and **Stephan von Haehling:** writing – review and editing.

## Conflicts of Interest

Anselm A. Derda received honoraria for lectures by AstraZeneca, Bayer, BMS, Boehringer Ingelheim and Lilly not related to this article; Emanuele Marzetti received consulting fees from Pfizer outside of the present work. This work was supported by PRACTIS –Clinician Scientist Program of Hannover Medical School, funded by the German Research Foundation (DFG, ME 3696/3‐1) to Anselm A. Derda. Wolfram Doehner reports consulting fees from Aimediq, Bayer, Boehringer Ingelheim, Boston Scientific, Lilly, Medtronic, Pfizer, Sanofi‐Aventis, Sphingotec, Vifor Pharma, travel support from Pharmacosmos, and research support to the Institute from EU (Horizon2020), German Ministry of Education and Research, German Center for Cardiovascular Research, Boehringer Ingelheim, from Vifor Pharma related to this trial, and ZS Pharma. Stefan D. Anker reports grants and personal fees from Vifor and Abbott Vascular, and personal fees for consultancies, trial committee work and/or lectures from Actimed, Amgen, Astra Zeneca, Bayer, Boehringer Ingelheim, Bioventrix, Brahms, Cardiac Dimensions, Cardior, Cordio, CVRx, Edwards, Farraday, Impulse Dynamics, Janssen, Novartis, Occlutech, Pfizer, Respicardia, Servier, Vectorious, and V‐Wave, and declares that he is named co‐inventor of two patent applications regarding MR‐proANP (DE 102007010834 and DE 102007022367), but he does not benefit personally from the related issued patents. Thomas Thum received honoraria for lectures/consulting from Bayer, Boehringer Ingelheim, Sanofi‐Genzyme, Takeda, Novo Nordisk, not related to this article. Thomas Thum filed and licensed noncoding RNAs as treatment targets and diagnostics. Thomas Thum is founder and CSO/CMO of Cardior Pharmaceuticals GmbH, a wholly owned subsidiary of Novo Nordisk A/S, not related to this article. Stephan von Haehling has been a paid consultant for and/or received honoraria payments from AstraZeneca, Bayer, Boehringer Ingelheim, BRAHMS, Chugai, Grünenthal, Helsinn, Hexal, Novartis, Novo Nordisk, Pharmacosmos, Respicardia, Roche, Servier, Sorin, and Vifor. Stephan von Haehling reports research support from Amgen, Boehringer Ingelheim, IMI, and the German Center for Cardiovascular Research (DZHK). All other authors declare no conflicts of interest.

## Data Availability

Data will be made available upon reasonable request.
